# Oxidative Processes and Xenobiotic Metabolism in Plants: Mechanisms of Defense and Potential Therapeutic Implications

**DOI:** 10.3390/jox14040084

**Published:** 2024-10-18

**Authors:** Caterina Vicidomini, Rosanna Palumbo, Maria Moccia, Giovanni N. Roviello

**Affiliations:** 1 Institute of Biostructures and Bioimaging, Italian National Council for Research (IBB-CNR), Area di Ricerca Site and Headquarters, Via Pietro Castellino 111, 80131 Naples, Italy; 2 Institute of Crystallography, Italian National Council for Research (IC-CNR), Strada Provinciale 35d, 9, Montelibretti, 00010 Rome, Italy

**Keywords:** plant metabolites, xenobiotic interactions, bioactive compounds, *Solanum aethiopicum*, metabolomics, drug metabolism, human health

## Abstract

Plants are continuously exposed to environmental challenges, including pollutants, pesticides, and heavy metals, collectively termed xenobiotics. These substances induce oxidative stress by generating reactive oxygen species (ROS), which can damage cellular components such as lipids, proteins, and nucleic acids. To counteract this, plants have evolved complex metabolic pathways to detoxify and process these harmful compounds. Oxidative stress in plants primarily arises from the overproduction of hydrogen peroxide (H_2_O_2_), superoxide anions (O_2_^•−^), singlet oxygen (^1^O_2_), and hydroxyl radicals (^•^OH), by-products of metabolic activities such as photosynthesis and respiration. The presence of xenobiotics leads to a notable increase in ROS, which can result in cellular damage and metabolic disruption. To combat this, plants have developed a strong antioxidant defense mechanism that includes enzymatic antioxidants that work together to eliminate ROS, thereby reducing their harmful effects. In addition to enzymatic defenses, plants also synthesize various non-enzymatic antioxidants, including flavonoids, phenolic acids, and vitamins. These compounds effectively neutralize ROS and help regenerate other antioxidants, offering extensive protection against oxidative stress. The metabolism of xenobiotic substances in plants occurs in three stages: the first involves modification, which refers to the chemical alteration of xenobiotics to make them less harmful. The second involves conjugation, where the modified xenobiotics are combined with other substances to increase their solubility, facilitating their elimination from the plant. The third stage involves compartmentalization, which is the storage or isolation of conjugated xenobiotics in specific parts of the plant, helping to prevent damage to vital cellular functions. Secondary metabolites found in plants, such as alkaloids, terpenoids, and flavonoids, play a vital role in detoxification and the defense against oxidative stress. Gaining a deeper understanding of the oxidative mechanisms and the pathways of xenobiotic metabolism in plants is essential, as this knowledge can lead to the formulation of plant-derived strategies aimed at alleviating the effects of environmental pollution and enhancing human health by improving detoxification and antioxidant capabilities, as discussed in this review.

## 1. Introduction

Understanding the interactions between plant metabolites and environmental xenobiotics, including pesticides, heavy metals, volatile organic compounds, and pharmaceuticals from treated wastewater and biosolids, is crucial due to their increasing exposure and potential impact [1]. A new approach in plant pharmacology aims to understand how pollutants interact with plants by studying plant pharmacokinetics (absorption, distribution, metabolism, and accumulation of xenobiotics) and pharmacodynamics (effects on plant enzymes and biochemical pathways). In fact, this dual framework enhances knowledge of xenobiotic behavior in plants, supports model development, and improves risk assessments for environmental and human health [1]. Plant metabolites, including alkaloids, polyphenols, flavonoids, saponins, and terpenes, are recognized for their bioactive properties and potential health benefits [2,3,4,5]. They play pivotal roles in plant defence, growth, and development, while recent research has also highlighted their significant interactions with xenobiotics, which encompass pharmaceutical drugs, environmental pollutants, and dietary compounds [6,7]. Notoriously, contaminants that impact human health have become a significant concern, leading to numerous investigations into methods for mitigating their harmful effects [8]. In this context, recent research has highlighted various biological roles of natural dietary compounds found in plants, which have shown promise in disease prevention and in countering chemically-induced cancer. The toxicity and carcinogenic effects of contaminants are primarily due to the mutagenic properties of reactive metabolites and the disruption of normal biological processes. Thus, effectively managing the metabolism of these hazardous substances is crucial for minimizing their adverse health impacts. Additionally, enhancing the elimination of contaminants from the body through metabolic processes is a valuable approach for reducing toxicity [8]. Maintaining balanced levels of reactive oxygen species (ROS) and reactive nitrogen species (RNS) is crucial for normal cellular function, a process known as redox homeostasis [9]. Key ROS include superoxide radicals (O_2_^•−^), hydrogen peroxide (H_2_O_2_), hydroxyl radicals (^•^OH), and singlet oxygen (^1^O_2_), while nitric oxide (^•^NO) is a primary RNS, all of which emerge as by-products of cellular metabolism under physiological and pathological conditions [10]. Enzymes such as cytochrome P450 (CYP), nitric oxide synthase (NOS), xanthine oxidase (XO), NADPH oxidase (NOX), cyclooxygenase (COX), and lipoxygenase (LOX) are central to the production of intracellular ROS [11,12] with NOX, composed of membrane-bound proteins gp91phox and p22phox, generating superoxide radicals by transferring electrons from NADPH to oxygen [13]. On the other hand, XO produces substantial ROS during the catalytic conversion of hypoxanthine and xanthine to uric acid [14], whereas the CYP family is involved in phase I xenobiotic metabolism and can produce ROS through reaction uncoupling [15]. NOS catalyzes the production of nitric oxide radicals during the conversion of L-arginine to citrulline [16], while COX and LOX enzymes oxidize arachidonic acid (AA) to produce ROS, which in turn stimulate NOX [17]. This review delves into the oxidative processes and xenobiotic metabolism in plants, highlighting how plants manage environmental challenges such as pollutants, pesticides, and heavy metals.

### 1.1. Highlights

- Xenobiotic defence mechanisms in plants: the study emphasizes how plants are equipped with intricate enzymatic (e.g., cytochrome P450, peroxidases) and non-enzymatic (e.g., flavonoids, vitamins C and E) antioxidant systems to defend against oxidative stress induced by xenobiotics like pollutants, pesticides, and heavy metals.
- Oxidative stress pathways: xenobiotic exposure elevates ROS such as hydrogen peroxide, superoxide anions, and hydroxyl radicals, which damage cellular components. Plants counteract this with antioxidants like catalase, superoxide dismutase, and glutathione.
- Therapeutic potential: the metabolic pathways in plants offer valuable insights into developing plant-based detoxification strategies that can mitigate the impact of environmental pollution and improve human health by harnessing plant metabolites.

### 1.2. Significance of the Study

- Environmental health insights: this research contributes to understanding how plants can manage the adverse effects of xenobiotics, providing strategies to mitigate the impacts of xenobiotic-based environmental pollution on plant and human health.
- Therapeutic applications: by exploring the xenobiotic metabolism in plants, the study opens up possibilities for developing plant-based remedies or enhancing the dietary use of plants to boost human health through their detoxification and antioxidant properties.
- Redox homeostasis and disease prevention: understanding how plants maintain redox homeostasis offers insights into preventing diseases caused by oxidative stress, such as cancer, highlighting the role of plant-based compounds in counteracting chemically-induced toxicity.
- Model for risk assessment: the dual focus on plant pharmacokinetics and pharmacodynamics enhances the ability to model and assess risks posed by environmental contaminants and develop new plant-based strategies for detoxification.

## 2. Oxidative Processes and Xenobiotic Metabolism by Plants

### 2.1. Environmental Challenges and Oxidative Stress in Plants

Plants face ongoing environmental challenges such as pollutants, pesticides, and heavy metals, collectively known as xenobiotics [18,19]. These substances induce oxidative stress by generating reactive oxygen species, which can harm cellular components such as lipids, proteins, and nucleic acids. To counteract this, plants have evolved intricate metabolic pathways that utilize different mechanisms to detoxify and process these harmful compounds [20,21]. Oxidative stress in plants primarily arises from the overproduction of H_2_O_2_, O_2_^•−^, ^1^O_2_, and ^•^OH, which are by-products of metabolic activities like photosynthesis and respiration [22,23]. Exposure to xenobiotics can significantly elevate ROS levels, causing cellular damage and disrupting metabolism. Plants employ a robust antioxidant defense system to mitigate oxidative damage, prominently featuring enzymatic antioxidants such as catalase (CAT), superoxide dismutase (SOD), and several types of peroxidases (POD) [24]. These enzymes collaborate to scavenge ROS and minimize their detrimental effects: SOD converts superoxide anions into hydrogen peroxide and oxygen, while CAT breaks down hydrogen peroxide into water and oxygen, thus reducing oxidative stress. Peroxidases utilize hydrogen peroxide to oxidize diverse substrates, further detoxifying ROS [24]. Additionally, plants produce numerous non-enzymatic antioxidants, such as flavonoids, phenolic acids, vitamin C, and vitamin E, which directly neutralize ROS, chelate metal ions, and regenerate other antioxidants, thereby providing comprehensive protection against oxidative stress [25].

### 2.2. Xenobiotic Metabolism in Plants

**Scheme 1:**
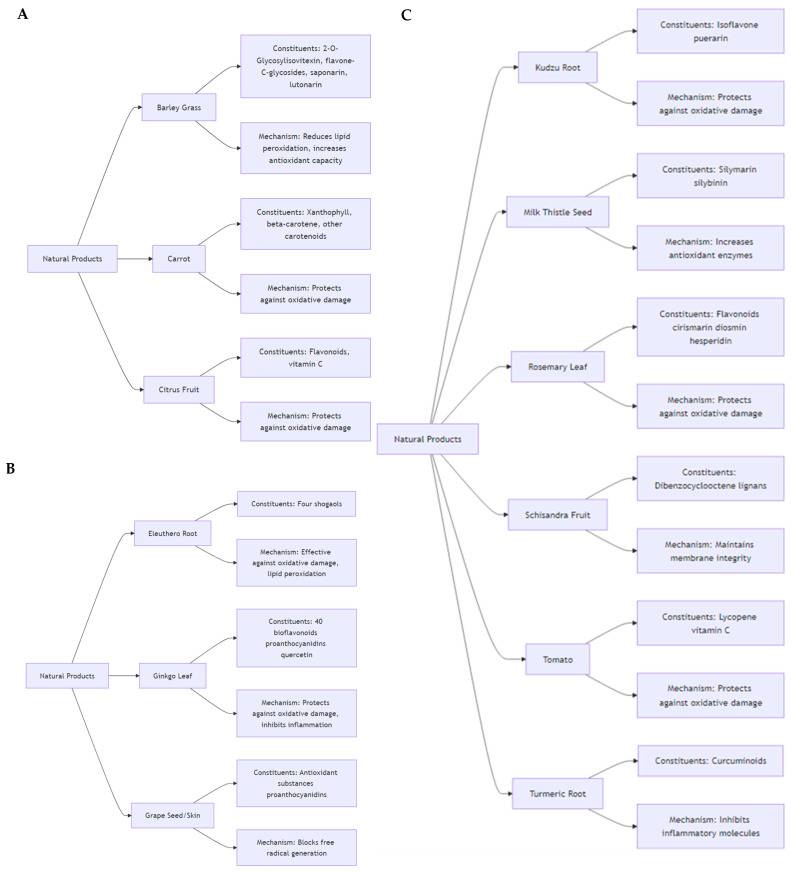
Flowcharts (**A**–**C**). Flowchart (**A**) describes the protective effects of a group of natural products, including barley grass, carrot, and citrus fruit, highlighting their antioxidant chemicals and mechanisms. Flowchart (**B**) reports on another group of natural products: eleuthero root, ginkgo leaf, and grape seed/skin, emphasizing their protective chemicals and actions against oxidative damage. Flowchart (**C**) shows the mechanisms of action for a further group of natural products, such as kudzu root, milk thistle seed, rosemary leaf, Schisandra fruit, tomato, and turmeric root, detailing their specific chemicals and how they inhibit inflammation and enhance antioxidant defenses.

Xenobiotics are metabolized in plants through a sequence of biochemical reactions known as xenobiotic metabolism, typically divided into three phases. Phase I involves oxidation, reduction, or hydrolysis of xenobiotics, often mediated by cytochrome P450 monooxygenases [26], which increase the compound’s reactivity and solubility. In Phase II, the reactive metabolites from Phase I are conjugated with endogenous molecules like glutathione, sugars, or amino acids by transferases such as glutathione S-transferases (GSTs), enhancing their solubility and facilitating detoxification and excretion. Phase III involves the transport of conjugated xenobiotics into vacuoles or extracellular spaces via ATP-binding cassette (ABC) transporters, preventing their interaction with cellular components and reducing toxicity [27]. Plant secondary metabolites, including alkaloids, terpenoids, and flavonoids, play crucial roles in xenobiotic metabolism and detoxification [28]. These compounds can modulate the activity of detoxification enzymes, bolster antioxidant defenses, and directly interact with xenobiotics to neutralize their toxic effects. For example, flavonoids can inhibit ROS production and enhance GST activity, while terpenoids, noted for their antioxidant properties, shield plant cells from oxidative damage induced by xenobiotics. Understanding the oxidative processes and xenobiotic metabolism in plants holds significant implications for human health, as many plants used in traditional medicine [29] contain bioactive metabolites that detoxify xenobiotics and alleviate oxidative stress (Table 1, Scheme 1) [30].

**Figure 1:**
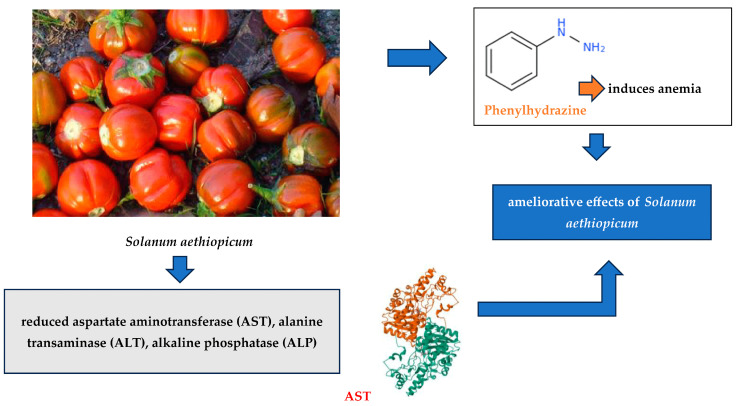
Diagram showing the ameliorative effects of *Solanum aethiopicum* (photo courtesy of Giovanni N. Roviello) on anemia induced by the xenobiotic compound phenylhydrazine. The 3D structure of AST is freely available at the link https://www.rcsb.org/structure/1AAM (accessed on 8 August 2024).

Integrating these plants into the diet or utilizing their compounds in pharmacology can enhance the body’s defenses against xenobiotics, potentially lowering the risk of diseases associated with oxidative stress and xenobiotic exposure. For example, the metabolites (especially flavonoids, saponins, and tannins) in leaf extracts of *Solanum aethiopicum*, whose fruits are widely used as a food source and in ethnomedicine, are believed to be responsible for the ameliorative effects on phenylhydrazine-induced anemia in rats. This demonstrates the plant’s potential in mitigating the toxic effects of phenylhydrazine, a known xenobiotic compound (Figure 1) [50].

The results of the mentioned study indicated a significant increase in packed cell volume, haemoglobin, and red blood cell counts in both male and female rats compared to other groups. Furthermore, the *S. aethiopicum* extract reduced levels of aspartate aminotransferase (AST, Figure 1), alanine transaminase (ALT), alkaline phosphatase (ALP), urea, creatinine, and chloride, supporting the use of *S. aethiopicum* leaf extracts as an effective anti-anaemic tonic with a wide margin of safety and potential hepato/reno-protective properties [50]. Moreover, the same plant was found to also have protective effects against sodium fluoride that notably reduces sperm quality, hormone levels, antioxidant concentrations, and overall body weight, while significantly increasing the levels of markers of oxidative stress. The same xenobiotic also leads to structural changes in the seminiferous tubules, including almost empty lumens and vascular hemorrhage. However, these adverse effects were mitigated by extracts from fruits of *Solanum aethiopicum* and Vitamin C [51]. Besides the specific effects of *Solanum aethiopicum* against xenobiotics, plants have evolved sophisticated mechanisms to manage oxidative stress and metabolize xenobiotics, employing a blend of enzymatic and non-enzymatic antioxidants alongside complex metabolic pathways [52,53]. These adaptations safeguard plants from environmental stresses and offer promising therapeutic avenues for improving human health by mitigating the adverse effects of xenobiotics.

## 3. The Role of Oxidative Imbalance in the Response to Environmental Pollutants

Oxidative stress occurs when there is an imbalance between pro-oxidants and antioxidants, resulting in cellular damage. Essentially, it happens when the production of free radicals exceeds the body’s capacity to neutralize them with antioxidants [54]. Free radicals, such as reactive oxygen species and reactive nitrogen species, are generated at a rate of approximately fifteen thousand per cell per day in healthy humans. These species include superoxide anions, hydroxyl radicals, hydrogen peroxide, singlet oxygen, and peroxyl radicals. While cellular ROS production is a well-documented phenomenon, their effects can be both beneficial and harmful under normal and pathological conditions. Interestingly, moderate levels of ROS are crucial for various cellular functions like proliferation, immune responses, metabolism, and survival, as they regulate signaling pathways [55,56]. For instance, mitochondrial ROS (mROS) play pivotal roles in erythroid progenitor cell proliferation, red blood cell formation, human fibroblast cell proliferation, vascular smooth muscle cell activation, and T-lymphocyte proliferation and activation. ROS levels increase in response to the platelet-derived growth factor (PDGF), essential for the growth of primary vascular smooth muscle cells (VSMCs) [57]. Inhibiting PDGF-induced ROS or enhancing catalase levels to scavenge ROS has been shown to reduce cell growth. Additionally, ROS regulate cell cycle progression, apoptosis, and cytokine and hormonal signaling through phosphoinositide-3-kinase (PI3K) [58] activation and downstream elements. Despite their critical roles in normal cellular functions, high ROS levels are cytotoxic. In particular, elevated ROS levels are associated with cancer cell proliferation, as cancer cells produce large amounts of ROS, particularly H_2_O_2_, required for mitogenic signaling. Loss of tumor suppressor genes and expression of oncogenes in a manner dependent on mROS have been observed in mouse embryonic fibroblast cells. Mutations in mitochondrial DNA (mDNA) encoding respiratory electron transport chain subunits lead to increased mROS levels, promoting tumorigenesis in mouse models and human and mouse cell lines. In heterozygous mice for mitochondrial transcription factor A (TFAM+/−), a stabilizing protein for mDNA, showed increased tumorigenesis in a mROS-dependent manner in intestinal carcinoma [59]. Free radicals act as signaling molecules at moderate concentrations, but their highly reactive nature necessitates strict regulation by the cellular antioxidant defense system to maintain redox balance. This balance is critical for normal cellular functioning and homeostasis. Consequently, biological processes generating ROS are coupled with various ROS scavenging mechanisms utilizing enzymatic and non-enzymatic antioxidants. As previously mentioned, enzymatic antioxidants like SOD, CAT, and glutathione peroxidase [60,61,62] constitute a primary defense mechanism.

### Environmental Pollutants and Their Impact on Oxidative Stress

**Figure 2:**
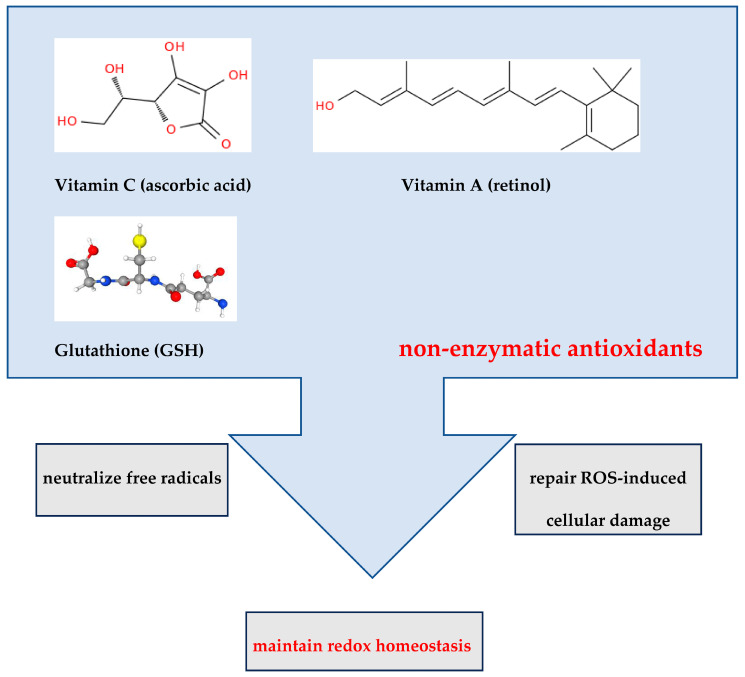
Vitamin C, Vitamin A, and Glutathione (GSH) are some of the main non-enzymatic antioxidants that play significant roles in maintaining redox homeostasis. The 3D structure of GSH is freely available at the link https://pubchem.ncbi.nlm.nih.gov/compound/448041#section=3D-Conformer (accessed on 8 August 2024).

**Figure 3:**
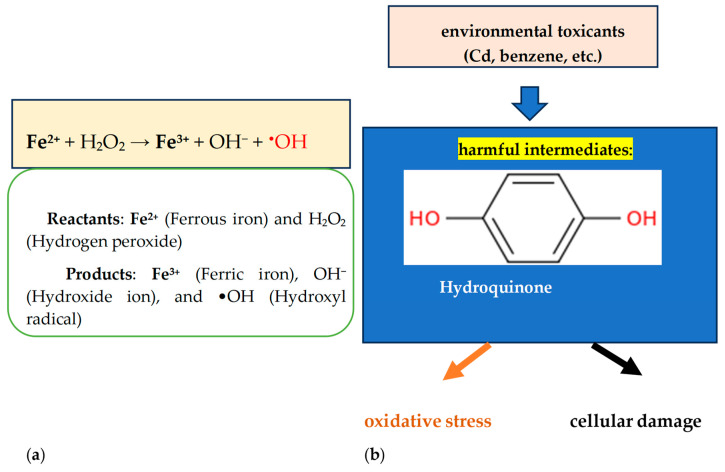
(**a**) Representation of the Fenton reaction and the production of hydroxyl radicals (^•^OH), which play a crucial role in the development of oxidative stress-related diseases and conditions; (**b**) diagram showing hydroquinone, a harmful intermediate produced after xenobiotic exposure, which leads to cellular damage and oxidative stress.

Non-enzymatic antioxidants, including vitamins C, and A, GSH (Figure 2), carotenoids, and flavonoids, also play significant roles in maintaining redox homeostasis. Vitamin C, a hydrophilic antioxidant, acts alone or synergistically with vitamin E, carotenoids, and antioxidant enzymes to neutralize free radicals [59]. On the other hand, vitamin E, a lipophilic antioxidant, effectively neutralizes lipid peroxyl radicals, while GSH, a tripeptide found abundantly in the nucleus, mitochondria, and cytoplasm, actively participates in ROS neutralization and repairs ROS-induced cellular damage. Despite these antioxidant systems, exposure to environmental toxicants can disrupt redox balance, leading to elevated ROS levels. This accumulation of ROS is a primary consequence of environmental toxicant exposure, causing cellular damage by impairing biomolecules and disrupting cell survival pathways. In particular, different environmental toxicants induce various biological effects by interfering with biological processes and targeting specific organs. Generally, ROS formation is a fundamental step in environmental toxicant-induced biological anomalies. As for the mechanisms of ROS generation, they vary depending on the toxicant’s chemical nature, dose, route, and duration of exposure. Environmental toxicants induce ROS formation by inhibiting the antioxidant defense system, initiating Fenton reactions (Figure 3a), causing mitochondrial dysfunction, and generating harmful chemical metabolites. Remarkably, heavy metals such as arsenic, chromium, cadmium, and lead inhibit SOD, CAT, GPx, and GSH, leading to redox imbalance. The above mentioned Fenton reaction (Figure 3a) [63,64,65], activated by heavy metals, generates free radicals through cascading reactions involving H_2_O_2_. Mitochondrial dysfunction, disturbed mitochondrial membrane potential (MMP), and production of harmful metabolites from volatile organic compounds also contribute to increased ROS levels. Exposure to environmental toxicants like cadmium and benzene can inhibit antioxidant enzymes, activate NADPH oxidase (which disrupts cellular homeostasis, promotes pathological conditions, and contributes to the development of various diseases), and produce harmful intermediates such as hydroquinone (Figure 3b), resulting in oxidative stress and cellular damage [59].

Given the diverse impact of environmental toxicants on oxidative stress and cellular damage, Table 2 presents a summary of some natural products that have been studied for their protective effects against such toxicity.

## 4. Phytochemicals as Free Radical Neutralizers in Combating Chemical-Induced Toxicity

### 4.1. Reproductive Health 

Reproductive health, a vital aspect of overall well-being, intricately linked to both individual and population health, is influenced by a multitude of factors, including environmental exposures, which can significantly impact developmental and reproductive processes. In this context, understanding how to mitigate the negative effects of environmental stressors is crucial. One of the emerging areas of research focuses on the role of phytochemicals as free radical neutralizers in combating chemical-induced reproductive toxicity. These natural compounds, found in a variety of plants, offer promising protective effects against oxidative stress caused by free radicals. In particular, advances in understanding developmental and reproductive biology have been significant, yet unraveling their intricate interaction with the environment remains complex. Key factors such as genetics, nutrition, temperature, and environmental influences all play critical roles in ensuring healthy development and reproduction.

#### 4.1.1. The Impact of Reactive Oxygen Species

Previous studies have elucidated the dual effects of reactive oxygen species levels on embryonic development, organismal growth, and reproductive systems. ROS are indispensable for early embryonic cell cycles in *Xenopus* embryos and facilitate rapid wound healing in embryos of *Drosophila* and zebrafish [79,80]. Additionally, ROS act as secondary messengers, modulating various signaling pathways during embryogenesis. However, heightened ROS levels have been linked to compromised embryo development and complications during pregnancy. In males, maintaining low ROS levels is essential for supporting crucial processes like sperm maturation, acrosome reaction, and sperm-oocyte fusion. Similarly, females require precisely regulated ROS levels for successful oocyte maturation and favorable pregnancy outcomes. Conversely, excessive ROS contributes to male infertility issues such as sperm mortality and female reproductive disorders such as polycystic ovary syndrome and endometriosis, often resulting in spontaneous abortions. Oxidative stress induced by environmental toxicants poses substantial risks to both development and reproduction, prompting increasing interest in plant-based interventions [81].

#### 4.1.2. The Role of Boerhavia Diffusa in Counteracting Toxic Effects in Drosophila

Traditional herbs like *Boerhavia diffusa Linn* show promise in reversing developmental and reproductive toxicity caused by substances like toluene in *Drosophila* larvae [81]. More in detail, a study including a preliminary phytochemical screening and HPTLC profiling of *B. diffusa* aqueous extract (BDAE) determined the LC50 of toluene, with a sublethal dose of 200 ppm being fixed for the investigation. Four doses of BDAE (25, 50, 100, and 200 mg/mL) were used, designated as low dose, medium dose 1, medium dose 2, and high dose. The parameters assessed included the larval period, pupal period, percentage of egg hatching, morphometric analysis of eggs, larvae, pupae, and adults, fertility, fecundity, lifespan, and levels of antioxidant enzymes such as catalase, glutathione-S-transferase, and superoxide dismutase. Overall, the results of this study showed that the phytochemical and HPTLC characteristics were consistent with pharmacopoeial standards. The LC50 of toluene was found to be 430 ppm, whereas BDAE at medium dose 2 and high dose significantly mitigated the adverse effects on reproductive and developmental parameters, including larval period, pupal period, percentage of egg hatching, morphometric characteristics of larvae, pupae, and adults, fertility, fecundity, and lifespan in *Drosophila*. Additionally, the extract significantly elevated the levels of antioxidant enzymes. Overall, *B. diffusa*, with its rich active ingredients, effectively prevented toluene-induced developmental and reproductive toxicity in *Drosophila*. This medicinal herb offers a promising solution for preventing reproductive and developmental toxicity induced by environmental toxins [81].

#### 4.1.3. Additional Phytochemicals and Their Impacts

**Figure 4:**
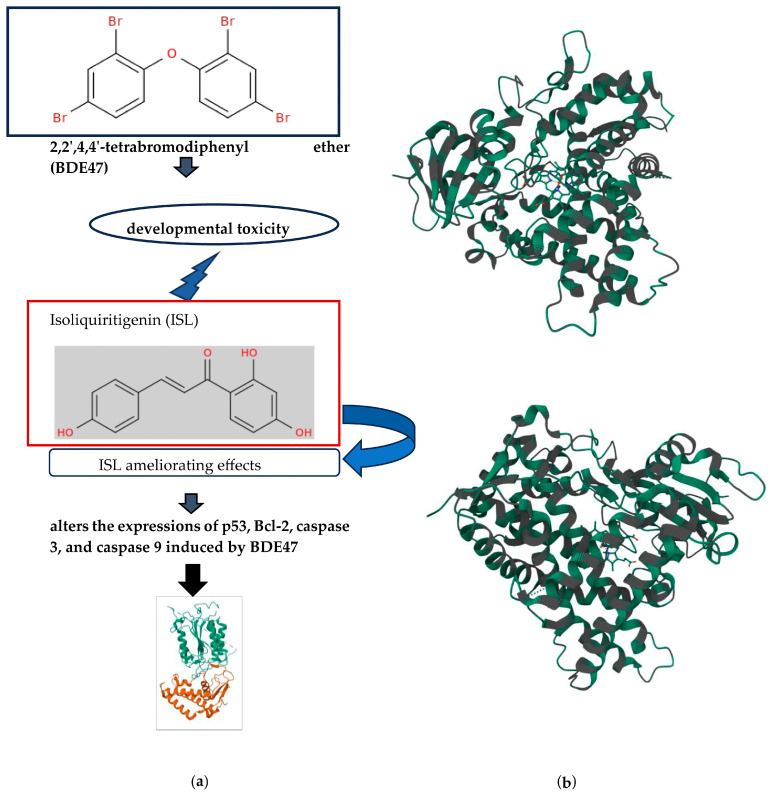
(**a**) Diagram illustrating the ameliorating effects of ISL, a natural chalcone-type flavonoid with antioxidant, anti-allergic, anti-inflammatory, anti-tumor, and estrogenic properties, against the developmental toxicity of BDE47, an abundant polybrominated diphenyl ether; the 3D structure of caspase 9 is freely available at the link https://www.rcsb.org/structure/3V3K (accessed on 8 August 2024) (**b**) 3D views of the human microsomal cytochrome P450 2E1 (CYP2E1) structure in complex with the inhibitor 4-methylpyrazole. CYP2E1 is involved in the monooxygenation of over 70 low molecular weight xenobiotic compounds. The structure can be freely accessed at https://www.rcsb.org/3d-view/3E4E/1 (accessed on 27 July 2024).

Isoliquiritigenin (ISL, Figure 4a) from *Glycyrrhiza uralensis* has demonstrated protective effects against developmental abnormalities induced by environmental chemicals in zebrafish embryos by enhancing antioxidant enzyme activities such as SOD and CAT [82].

Specifically, the study focused on 2,2′,4,4′-tetrabromodiphenyl ether (BDE47, Figure 4a), the most abundant polybrominated diphenyl ether (PBDE) found in biological samples. BDE47 has a strong tendency to bioaccumulate and potentially endangers mammalian development through oxidative stress. ISL [83], an emerging natural chalcone-type flavonoid, possesses various biological and pharmacological properties, including antioxidant, anti-allergic, anti-inflammatory, anti-tumor, and estrogenic activities. Interestingly, a recent study explored the antioxidant effect of ISL on ameliorating developmental anomalies induced by BDE47. Zebrafish (*Danio rerio*) [84] embryos were exposed to BDE47 (1 and 10 μM) and/or ISL (4 μM) for 4 to 120 h post-fertilization, and the morphology, development, behavior, oxidative stress status, and related gene expressions were assessed. The results showed that BDE47 caused dose-dependent growth retardation and deformities, including delayed hatching, spinal curvature, reduced body length, increased death rate, aberrant behaviors, and impaired dark-adapted vision, which were significantly mitigated by ISL. Furthermore, ISL ameliorated excessive ROS accumulation and altered the expressions of apoptosis-related genes p53, Bcl-2, caspase 3, and caspase 9 induced by BDE47. This suggests that ISL protected zebrafish from the developmental toxicity of BDE47 by inactivating programmed apoptosis and activating antioxidant signaling pathways. Overall, developing ISL as a dietary supplement might be a promising preventive strategy for ameliorating developmental toxicity induced by environmental pollutants [82].

#### 4.1.4. Mitigating Ovarian and Uterine Toxicity by *Calliandra portoricensis*

*Calliandra portoricensis* (CP) is an herb popular in Nigeria that has proven effective in alleviating ovarian and uterine toxicity in rats exposed to carcinogens due to its anti-inflammatory and antioxidant properties [85]. In fact, the study of Adefisan et al. addressed the traumatic impact of infertility resulting from reproductive impairment within families. More in detail, the ovarian and uterine toxicity induced by benzo[a]pyrene (BaP) and N-methyl nitrosourea (NMU) was examined, along with the mitigating effects of *Calliandra portoricensis* [85]. The aim was to shed light on folk medical claims, uncover lost knowledge, advance natural chemopreventive agents, and present new evidence regarding CP’s effects. Although CP is known for its anticonvulsant, antidiarrheal, antipyretic, antirheumatic, and analgesic properties, the mentioned study demonstrated also its potential role in mitigating reproductive toxicity from chemical exposures. The findings confirmed and expanded on CP’s ability to reduce toxic responses resulting from oxidative damage and inflammatory reactions associated with NMU and BaP exposure. Therefore, the development of phytochemicals derived from CP may offer a natural therapy against chemical toxicities for individuals inadvertently exposed, thereby promoting human health and reproductive well-being [85].

#### 4.1.5. Further Plant-Based Studies and Their Findings

Other botanical studies highlight sweet potato (*Ipomoea batatas* L. Lam.) extract, which has boosted antioxidant levels and reproductive health in male rats exposed to bisphenol A, and *Mentha spicata* extract, which has mitigated nicotine-induced oxidative stress in rat brains and testes by restoring antioxidant enzyme levels [86]. More in detail, the study focused on sweet potato [87], known as “Shakarqandi” in Pakistan, an important root vegetable traditionally used for its aphrodisiac, antiprostatic, anti-inflammatory, antidiabetic, cardiotonic, and anticancer properties, aimed to gauge the aphrodisiac potential of *Ipomoea batatas* ethyl acetate (IPT-EA, IPA-EA) and methanol (IPT-M, IPA-M) extracts from tuber and aerial parts, respectively, through behavioral and biochemical tests, and to assess their protective role in bisphenol A (BPA)-induced gonadotoxicity at a dose of 300 mg/kg in male Sprague Dawley rats. Phytochemical analysis was conducted both qualitatively and quantitatively, assessing total phenolic and flavonoid content (TPC and TFC) and using high-performance liquid chromatographic (HPLC-DAD) fingerprinting. Antioxidant profiling employed multimode in vitro assays, biochemical validation included semen characteristics and levels of testosterone, follicle-stimulating hormone (FSH), luteinizing hormone (LH), and estradiol, while gonadoprotective ability was assessed using the comet assay and histomorphological examination of testes. Overall, qualitative analysis of sweet potato extracts confirmed the presence of phenols, flavonoids, tannins, anthocyanins, saponins, coumarins, terpenoids, and betacyanins. Quantitative analysis revealed that the IPA-EA extract had the highest TPC (304.32 ± 7.20 μg gallic acid equivalents/mg dry extract) and TFC (214.77 ± 4.09 μg quercetin equivalents/mg dry extract). IPT-EA yielded maximum rutin (7.3 ± 0.12 μg/mg dry extract) and myricetin (2.7 ± 0.14 μg/mg dry extract), while IPA-EA and IPA-M extracts yielded the highest levels of caffeic acid (4.05 ± 0.22 and 1.92 ± 0.17 μg/mg dry extract, respectively) in HPLC-DAD analysis (Table 3).

The extracts were found to enhance sexual excitement, improve semen quality, and elevate levels of testosterone, FSH, LH, and estradiol, effectively mitigating the toxic effects of BPA. The levels of endogenous antioxidant enzymes (CAT, SOD, POD, and GSH) were restored, and nitric oxide abundance was minimized. Significant stimulation in sexual behavior, amelioration of toxicity symptoms, increased sperm production, enhanced viability, elevated levels of gonadal hormones, maintained endogenous enzyme activity, genoprotection, and improved testicular histology endorsed *I. batatas* as an effective aphrodisiac and gonadoprotective agent [86]. Apart from the above example, compounds like ellagic acid [88] and ferulic acid [89] have shown protective effects against testicular damage and oxidative stress caused by arsenic in mice, while taxifolin has protected fetal male rats from reproductive toxicity induced by di-n-butyl phthalate [90]. Treatment with the plant hormone 24-epibrassinolide [91] restored redox balance and enhanced health outcomes in ethanol-exposed zebrafish embryos, and the phytomolecules thymoquinone and curcumin have demonstrated beneficial effects in mitigating acrylamide-induced developmental issues in *Drosophila* by regulating ROS levels. Allicin derived from garlic [92] reversed lead-induced cognitive deficits in rats by managing oxidative stress [67]. These studies collectively underscore the potential of phytochemicals as safe and effective supplements for combating oxidative stress-related developmental and reproductive toxicity due to environmental pollutants. 

### 4.2. Phytochemicals in Liver and Renal Protection

**Figure 5:**
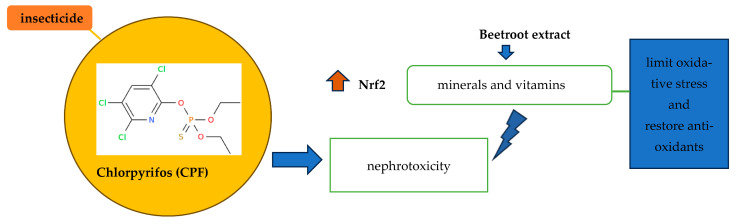
Diagram showing how *Beta vulgaris* L. (beetroot), known for its minerals and vitamins, counteracts chlorpyrifos (CPF) nephrotoxicity by restoring antioxidants and limiting oxidative stress. Beetroot products have effects that increase nuclear factor erythroid 2-related factor 2 (Nrf2), which is crucial in promoting the expression of defensive enzymes.

The liver, a vital organ, performs crucial functions such as xenobiotic metabolism, immune response facilitation, digestion, and vitamin storage. Hepatocytes, comprising 60–80% of liver cells, are primarily responsible for metabolic and detoxification activities. Oxidative stress is a pivotal pathological mechanism underlying liver damage, including conditions like alcoholic liver disease, steatosis, and cirrhosis. Within hepatocytes, ROS predominantly arise from cytochrome P450 enzymes in mitochondria and the endoplasmic reticulum. Environmental toxins induce hepatic injury through oxidative stress pathways, highlighting the importance of managing redox balance to mitigate liver toxicity. Recent research emphasizes the protective role of plant-derived compounds against chemical-induced liver damage. For instance, exposure to the above-mentioned BPA diminishes antioxidant enzyme activity while increasing lipid peroxidation, DNA damage, and markers of hepatotoxicity like AST, ALP, and ALT. In contrast, the administration of *Quercus dilatata* Lindl. extracts has shown potential in alleviating the bisphenol A-induced liver injury [93]. Moreover, curcumin (from *Curcuma longa*) has been effective in ameliorating insulin resistance and oxidative stress induced by bisphenol A in HepG2 cells [59]. Ethanol consumption exacerbates liver damage by increasing ROS production during metabolism. Rats supplemented with Green *Capsicum annum* exhibited protective effects against ethanol-induced hepatotoxicity by enhancing the antioxidant defense system [94]. Purple potato (*Solanum tuberosum*) [95] extracts similarly protected against ethanol-induced liver damage by suppressing CYP2E1 (Figure 4b) expression. Additionally, a combination of curcumin and baicalin effectively ameliorated ethanol-induced liver damage by activating the Nrf2/HO-1 pathway [59]. Carbon tetrachloride (CCl_4_), a potent hepatotoxic agent [96,97,98], disrupts hepatic redox homeostasis by inducing CYP2E1. However, co-administration of *Citrus aurantium* ethyl acetate extract, *Syzygium samarangense* methanol extract, and *Aerva javanica* ethanolic extract effectively attenuated CCl4-induced hepatotoxicity by maintaining redox balance [99,100,101]. Coumarins such as umbelliferone and daphnetin protected against CCl_4_-induced liver damage by activating the Nrf2 pathway and enhancing cytoprotective antioxidant systems [102]. *Commelina nudiflora* supplementation also defended against CCl_4_-induced liver injury by modulating oxidative stress markers and inflammation, thereby preserving liver morphology [103]. Aluminum (Al) exposure increases ROS production in the liver, leading to oxidative stress-related liver injury. In this context, *Nigella sativa* oil [104,105,106], rich in phenolics, flavonoids, and tannins, has shown protective effects against aluminum-chloride-induced liver injury in rats. Similarly, *Monodora myristica* and *Terminalia arjuna* extracts have mitigated oxidative stress and improved liver health in response to aluminum toxicity [59]. Cadmium (Cd), another toxic metal, induces oxidative stress in the liver, causing lipid peroxidation, mitochondrial dysfunction, and apoptosis. Supplementation with *Vernonia amygdalina* acetonic extract [107], *Jessiaea nervosa* aqueous extract [108], and *Terminalia arjuna* bark aqueous extract [109] attenuates Cd-induced liver injury by restoring antioxidant levels and reducing lipid peroxidation. *Rosa persica* supplementation enhances metallothionein content and reduces oxidative stress in Cd-exposed organisms, thereby improving liver function. Arsenic exposure induces oxidative stress and oxidative DNA damage in the liver, promoting hepatocarcinogenesis. In this frame, sulforaphane supplementation activates the Nrf2 pathway via PI3K/Akt signaling, exhibiting hepatoprotective effects against arsenic-induced liver injury [59]. Overall, phytochemicals have emerged as promising preventive agents, enhancing antioxidant levels and supporting normal liver function in the face of toxic insults. Free radicals play a crucial role in xenobiotic-induced toxicity, affecting not only the liver but also renal function. Thymoquinone, found in *Nigella sativa* oil, counters As and Cd-related renal damage by restoring antioxidant enzymes like SOD, CAT, and GSH, curbing lipid peroxidation (LPO), and reducing apoptosis in renal tissue. Due to the lack of a specific excretory pathway, Cd accumulates in kidneys, harming proximal tubules in particular. Flavocoxid, a blend of natural plant-derived compounds rich in polyphenols, mitigates Cd-linked kidney lesions through its antioxidant properties. Ferulic acid, from curcumin, mitigates Cd renal damage in Wistar albino rats by inhibiting Cd accumulation, elevating antioxidant enzymes (TTH, TAC, SOD, CAT, GPx, TSH, and GSH), and controlling stress and inflammatory markers. The oxidative DNA damage monitored by the marker 8-OxodG, elevated in As-exposed C57BL/6 mice, was reduced with black raspberry. *Beta vulgaris* L. (beetroot), globally recognized for its minerals and vitamins, counters chlorpyrifos (CPF, Figure 5) nephrotoxicity by restoring antioxidants and limiting oxidative stress. Sodium dichromate and lithium carbonate increase renal markers (creatinine and blood urea nitrogen, BUN), LPO, reduce antioxidants, and cause DNA fragmentation [59]. *Opuntia ficus indica* [110,111,112,113] with its antioxidants, protects against renal damage from lithium carbonate and sodium dichromate [111,113]. Remarkably, the study of Ncigi et al. examined the effectiveness of *Opuntia* cladodes extract in mitigating liver damage caused by the organophosphorous insecticide chlorpyrifos in mice [112]. Liver damage was assessed by measuring liver weight and analysing various biochemical parameters, including alanine aminotransferase, aspartate aminotransferase, alkaline phosphatase, lactate dehydrogenase, cholesterol, and albumin in serum using spectrophotometric methods. The experimental design spanned 48 h and involved six different treatments with six mice each: (1) control, (2) 10 mg/kg (body weight, b.w) CPF, (3) 10 mg/kg (b.w) CPF with 100 mg/kg (b.w) cactus (*Opuntia*), (4) 150 mg/kg (b.w) CPF, (5) 150 mg/kg (b.w) CPF with 1.5 g/kg cactus, (6) 1.5 g/kg cactus. Both CPF and cactus were administered orally via gavage. The findings indicated that CPF significantly impacted all measured parameters. However, when CPF was administered with cactus extract, a recovery in all parameters was observed. Conversely, cactus alone did not alter the studied parameters. These results suggest that CPF is hepatotoxic and that *Opuntia ficus indica* stem extract offers protective effects against liver damage induced by this organophosphorous pesticide [112].

Mercury triggers renal oxidative stress and acute failure. *Juglans sinensis* extract prevents mercury chloride damage in rabbits by bolstering antioxidants [114]. *Eruca sativa* seed extract counters Hg-induced renal toxicity [59,115]. Selenium-induced Wistar rat hepatic and renal injuries are resolved by curcumin; plant extracts and metabolites alleviate oxidative stress, inflammation, and renal dysfunction caused by environmental toxicants. 

### 4.3. Neuroprotection and Environmental Toxins

Neurons are particularly susceptible to irreversible damage, profoundly affecting their structure and function, leading to the onset and progression of debilitating neurological disorders such as Alzheimer’s disease [116,117,118,119,120], Parkinson’s disease [121], and amyotrophic lateral sclerosis (ALS) [122,123]. These conditions pose significant global health challenges [124,125], ranking second only to cardiovascular diseases in terms of mortality, affecting countless individuals worldwide. The multifaceted nature of neurodegenerative diseases involves a complex interplay of genetic predispositions, lifestyle factors, and exposure to environmental agents. Key hallmarks of neurodegenerative diseases include cognitive impairment, dementia, motor dysfunction (such as ataxia), and speech difficulties. These symptoms manifest gradually over time and are exacerbated by increased oxidative stress, which plays a pivotal role in disease pathogenesis [126]. Neurons, being highly specialized cells with high oxygen consumption and relatively low levels of antioxidants, are particularly vulnerable to oxidative damage. This imbalance leads to impaired protein dynamics, aggregation of misfolded proteins, and ultimately, neuronal cell death. Environmental chemicals can breach the protective blood-brain barrier, directly affecting brain health through various mechanisms. These include mitochondrial dysfunction, oxidative stress induction, disruption of neurotransmitter signaling, and epigenetic modifications, all of which contribute to the development and progression of neurological disorders. In response to these challenges, there is growing interest in exploring plant-based interventions as potential therapeutic strategies against neurotoxicity induced by environmental toxins. Plant-derived compounds have demonstrated promising neuroprotective effects by restoring redox homeostasis, enhancing antioxidant defenses, and mitigating oxidative damage to neural cells [59,127,128]. For instance, different studies have highlighted the protective roles of various herbal extracts and phytochemicals, such as kolaviron [129], *Mentha spicata* [130], *Morus alba* [131], and mangiferin [59,132]. These products have been shown to attenuate oxidative stress, reduce neuroinflammation, and preserve neuronal function in preclinical models. 

Thus, while oxidative stress remains a critical factor in the pathogenesis of various diseases, including neurodegenerative ones [122,125,126,127,133], plant-based interventions offer potential avenues for therapeutic development by targeting underlying mechanisms of toxicity and enhancing resilience against environmental insults (Table 4).

Continued research in this area holds promise for advancing novel treatments aimed at mitigating the burden of different socially-relevant disorders worldwide. Even natural substances intended to boost antioxidant levels should be used with caution, as excessive intake may pose risks; for example, research has indicated that high doses of beta-carotene can increase the risk of lung cancer, particularly in smokers or former smokers, despite its availability in multivitamins [103].

### 4.4. Variability in Plant Metabolite-Xenobiotic Interactions

Although limited research specifically addresses how interactions between plant metabolites and xenobiotics may vary across different plant species, environmental conditions, or types of xenobiotics, it is essential to critically analyse these factors for a comprehensive understanding of plant-xenobiotic interactions. The interaction between plant metabolites and xenobiotics encompasses various metabolic processes that modify xenobiotics to diminish their toxicity and facilitate their removal. To enhance their solubility and decrease toxicity, xenobiotics are often conjugated with more polar molecules, including sugars, amino acids, organic acids, or glutathione. Specifically, plants frequently incorporate sugar moieties into xenobiotics using enzymes like UDP-O-glucosyltransferases and UDP-*N*-glucosyltransferases, which results in the formation of glycoside derivatives [134]. These glycosylated compounds may then undergo further modifications, such as acylation with malonic acid via O- or N-malonyltransferases, which improves their stability and solubility. Additionally, glutathione S-transferases play a significant role in this interaction by conjugating xenobiotics with reduced glutathione. This process generates glutathione S-conjugates that contribute to detoxifying the xenobiotics, making them less harmful to the plant. Various isoforms of glutathione transferases possess different substrate specificities, leading to distinct metabolic outcomes based on the specific plant species and their enzyme profiles. In plants, the resulting conjugated xenobiotics are usually sequestered in vacuoles or associated with insoluble structures like lignin and polysaccharides, given that plants do not have an active excretory system. This sequestration helps reduce the mobility and potential toxicity of xenobiotics within the plant. The diversity in conjugated metabolites can vary widely among different plant species, illustrating the complexity of these interactions and the ways in which plants adapt to chemical stressors in their environment. Gaining insight into these interactions is essential for understanding the ecological consequences of xenobiotic exposure and how various plants manage chemical challenges [134]. In the study conducted by Kolb and Harms, the authors focus on the metabolism of fluoranthene across various plant cell cultures, identifying significant differences in metabolic rates among the species examined. The findings indicate that specific plant species, such as *Rosa* sp. Paul’s Scarlet, exhibited remarkably high rates of fluoranthene metabolism compared to others. Additionally, the metabolic pathways activated in response to xenobiotics may vary with different chemical structures and functional groups. The study highlights the limited metabolic conversion of fluoranthene in comparison to other compounds like pentachlorophenol or 4-n-nonylphenol, suggesting that structural differences in these xenobiotics lead to varying enzymatic responses in plants [135]. Remarkably, the effectiveness of phytoremediation in removing or neutralizing xenobiotics from the environment depends heavily on various environmental conditions such as temperature, humidity, and sunlight, which can limit its application to specific areas [136]. Additionally, when xenobiotic concentrations are particularly high, they may become toxic to the plants themselves, affecting their capacity for remediation. Moreover, this technique typically requires a large amount of land to accommodate the necessary plant growth for effective treatment [136]. Research has shown that different xenobiotic compounds can exhibit varying degrees of toxicity and metabolic complexity, affecting how plants respond to these stressors. Some xenobiotic compounds, such as acetaminophen, are more easily metabolized by plants. Acetaminophen, commonly found in agroecosystems due to the reuse of treated wastewater, can be quickly conjugated with molecules like glutathione, glucuronic acid, or sulphate, allowing plants to process it efficiently through established metabolic mechanisms [137]. On the other hand, heavy metals such as cadmium and lead are highly toxic and can generate reactive oxygen species that lead to oxidative stress in plants. Research shows that different xenobiotics like Cd and Pb can affect plants in varying ways, particularly in terms of toxicity and oxidative stress. In mulberry plants (*Morus alba*) exposed to Cd and Pb stress (100 and 200 μmol L⁻^1^), both metals degraded chlorophyll and reduced photosynthesis, but Cd caused more severe damage overall. Cd stress led to higher production of ROS and greater oxidative damage than Pb, resulting in more pronounced reductions in photosynthetic activity. Pb, while still harmful, caused less disruption to electron transport and energy dissipation, making Cd the more challenging xenobiotic for the plant to handle [138]. *Thlaspi caerulescens* (alpine pennycress), a well-known hyperaccumulator of heavy metals, demonstrated a markedly different response compared to nonaccumulator species such as alfalfa (*Medicago sativa*), radish (*Raphanus sativus*), and lettuce (*Lactuca sativa*). Specifically, *T. caerulescens* showed superior resistance to the phytotoxic effects of Cd and Zn, and it was able to accumulate these metals at much higher concentrations within its tissues compared to the nonaccumulator plants. However, for Cu, *T. caerulescens* was less effective in accumulating it compared to alfalfa, indicating that its capacity to handle different types of heavy metals varies. This variation in metal accumulation abilities across species highlights that different plants possess distinct physiological and metabolic mechanisms to cope with xenobiotics, depending on their genetic traits and enzyme systems. These findings are supported by the significant differences in root elongation inhibition and metal translocation observed in the study, underscoring that species-specific traits greatly influence how plants deal with toxic compounds in the environment. Thus, this study confirms that plants vary significantly in their ability to handle different xenobiotics, with some species like *T. caerulescens* being particularly adept at accumulating certain metals like Cd and Zn, while struggling with others like Cu [139].

### 4.5. Differential Plant Responses to Xenobiotics

**Scheme 2:**
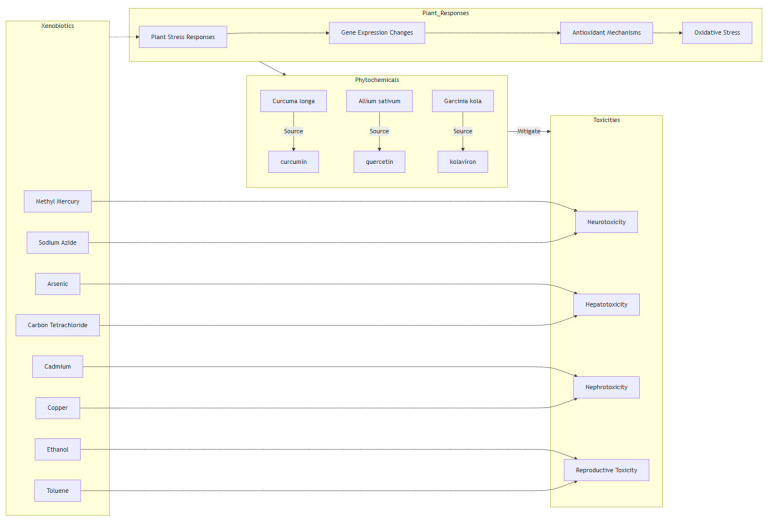
This diagram illustrates the intersection of plant metabolites, xenobiotics, and plant responses, showcasing the complex interactions where phytochemicals mitigate the adverse effects of harmful substances. It highlights how plants adapt through stress responses and gene expression changes, enhancing their resilience against environmental pollutants.

The xenobiotics can be organized into distinct categories based on their primary effects and the corresponding plant mechanisms involved in their amelioration. For neurotoxicity, examples include sodium azide, and methyl mercury, which induce oxidative stress, leading to neuronal cell death and cognitive dysfunction (Scheme 2). Phytochemicals such as kolaviron and mangiferin have shown protective effects through antioxidant activity and anti-inflammatory properties. Regarding the hepatotoxicity of bisphenol A, carbon tetrachloride, and arsenic cause increased lipid peroxidation and decreased antioxidant enzyme levels, resulting in liver damage. Phytochemicals like curcumin, thymoquinone, and those extracted from *Citrus aurantium* exhibit hepatoprotective effects by enhancing antioxidant defenses and reducing oxidative stress markers. For nephrotoxicity, copper, arsenic, cadmium, and lead disrupt renal function by inducing oxidative stress and inflammation. Protective compounds such as quercetin, taxifolin, and catechin help restore antioxidant balance and mitigate kidney damage. Regarding developmental and reproductive toxicity, xenobiotics such as toluene, ethanol, and nicotine can lead to developmental delays and reproductive issues via oxidative stress and hormonal disruption. Phytochemicals like those from *Boerhavia diffusa* and *Mentha spicata* help counteract these effects through their antioxidant properties. Finally, in the context of pulmonary toxicity, agents like paraquat and benzo(a)pyrene increase oxidative stress in lung tissues. Protective phytochemicals such as quercetin and procyanidin B2 have shown efficacy in reducing ROS levels and inflammation [59]. Understanding the impact of different dosages is crucial in evaluating plant responses, as the effects can vary significantly based on the concentration of the xenobiotic used. Although data on the relationship between varying xenobiotic doses and plant responses are limited, the study by Yang et al. provides valuable insights [140]. A bioassay was conducted to evaluate the effects of cycloxaprid on rice seedlings and third instar insects. To prepare cycloxaprid, it was initially dissolved in dimethylformamide (DMF) and then diluted in distilled water with 0.05% Triton-100 to create various concentrations. The bioassay determined the low dose (LC15) and high dose (LC85) for treatment, with the calculated LC15 being 0.44 μg mL⁻^1^ (95% confidence interval: 0.31–0.61) and the LC85 measuring significantly higher at 19.86 μg mL⁻^1^ (95% confidence interval: 13.63–28.95). In the experimental setup, four rice seedlings were immersed in each concentration for 15 s before being placed in a controlled environment, with mortality assessments conducted after 48 h. Under the high-dose condition, 4807 unigenes were differentially regulated, showing 1743 upregulated and 3063 downregulated. Conversely, the low-dose treatment affected 2020 unigenes, with 918 upregulated and 1122 downregulated. This variation in gene expression indicates that the organisms activated different stress response pathways based on the levels of cycloxaprid exposure.

Notably, 306 unigenes exhibited contrasting regulation patterns in response to high and low doses. The research also examined several gene families related to detoxification, including cytochrome P450s, glutathione S-transferases, and ATP-binding cassette transporters. In the case of P450s, ten unigenes showed increased expression under the high-dose treatment, with six belonging to the CYP6 family, highlighting their significant role in detoxification. A specific delta-GST unigene demonstrated a remarkable 66.64-fold increase in expression under low-dose treatment, in contrast to a modest 2.41-fold increase in a microsomal GST under high-dose conditions, indicating differing regulatory responses to the doses. Additionally, one acetylcholinesterase unigene was notably upregulated (9.46-fold) in the high-dose treatment, suggesting its potential involvement in the insect’s resistance mechanisms against cycloxaprid. Heat shock proteins (Hsps) also showed differential expression; the levels of two Hsp70 unigenes and two small Hsp unigenes rose more than two-fold in the high-dose treatment relative to the control, while they decreased in response to the low dose. Overall, these results reveal that varying doses of xenobiotic (cycloxaprid) trigger distinct responses in both plants and insects, emphasizing the intricate regulatory mechanisms governing detoxification and stress responses in *Sogatella furcifera* [140]. The complexity of plant responses is further illustrated by research involving *Medicago sativa* plants inoculated with *Sinorhizobium meliloti*, which demonstrated that cadmium and fluoranthene notably impacted nodulation and gene expression [140]. In this investigation, alfalfa was grown for 14 days in a mineral medium containing different concentrations of these xenobiotics. Key parameters measured included the number of root nodules, fresh weight of shoots and roots, and gene expression assessed through mRNA differential display techniques. The results showed that both cadmium and fluoranthene significantly decreased the number of root nodules before any visible signs of plant damage occurred. The effective concentration (EC_50_) for cadmium affecting nodulation was determined to be 5.8 µM, while fluoranthene exhibited an EC_50_ of 2.5 µg cm^2^. Notably, nodulation was found to be ten times more sensitive to these pollutants compared to shoot and root biomass; for instance, the EC_50_ for root fresh weight due to cadmium was over 20 µM, and more than 35 µg cm^2^ for fluoranthene. Regarding gene expression, the study identified 37 differentially displayed transcripts in response to the xenobiotics. One transcript, DDMsl, was confirmed to be downregulated in the presence of both cadmium and fluoranthene. In control plants inoculated with rhizobia, DDMsl expression was 2.5–3 times higher than in non-inoculated plants. However, this expression was significantly diminished by fluoranthene and completely suppressed by cadmium, with an EC_50_ for DDMsl expression of 5.9 µM. Sequence analysis of DDMsl indicated a 50% similarity to a copper-transporting ATPase from *Arabidopsis thaliana*. Another transcript, DDMs2, also showed down-regulation in plants treated with xenobiotics, revealing a 50% decrease in expression and demonstrating a strong homology (82% similarity) to the cytoplasmic 60S ribosomal protein L18 from *Arabidopsis thaliana* [140]. These findings suggest that nodulation is more susceptible to environmental stressors than overall plant growth, indicating that the initial stages of root nodule formation may be especially vulnerable to contaminants, potentially compromising nitrogen fixation and plant health. The differential expression of genes like DDMsl and DDMs2 in response to xenobiotics implies that these transcripts may be vital for plant stress responses and adaptation to heavy metals and organic pollutants. Gaining insights into these molecular responses can aid in formulating strategies to enhance plant resilience against environmental contaminants. The calculated EC_10_, EC_50_, and EC_90_ values are crucial for understanding how varying concentrations of pollutants affect plant physiology, which is essential for environmental risk assessments and developing regulations concerning heavy metal and hydrocarbon pollution in agricultural soils. Overall, this study underscores the considerable negative effects of xenobiotics on plants and highlights how different concentrations can distinctly impact nodulation and gene expression [140].

## 5. Conclusions

Human activities have significantly damaged the environment, resulting in widespread exposure to harmful xenobiotic chemicals [141]. Both short-term and long-term contact with these substances adversely affects living organisms, including humans, leading to negative impacts on health and well-being. Research over the past century has primarily focused on how these chemicals influence various organisms, with a recent shift towards a more detailed understanding of toxicity aimed at reducing harmful effects. The clinical assessment of phytocompounds that ameliorate xenobiotic-induced diseases, particularly drug-induced liver toxicity, highlights the potential and efficacy of herbal antioxidants. Many natural products, especially those used in traditional medicine in regions like Caucasus [3,29], China and India, have garnered significant interest for their therapeutic properties against liver disorders. Compounds rich in triterpenes, flavonoids, and polyphenols are increasingly recognized for their hepatoprotective effects in experimental liver injury models. These natural products are believed to provide protection primarily through their antioxidant properties, which help eliminate free radicals and mitigate reactive oxygen species damage to cellular structures. The clinical evaluation of specific compounds such as silymarin, resveratrol, curcumin, and ginkgo biloba highlights their mechanisms of action, including cell regeneration, cytoprotection, and modulation of various cytochrome P450 isoforms. For instance, silymarin from milk thistle has been shown to protect against drug-induced hepatotoxicity in various animal models and exhibits antioxidant and cell-regenerative properties, with clinical trials suggesting that silymarin can significantly reduce liver damage in patients with chronic liver diseases, with minimal side effects reported. Resveratrol, a polyphenol found in grapes, has been linked to chemoprevention and protection against liver damage induced by substances like acetaminophen, while curcumin, derived from turmeric, exhibits potent antioxidant and anti-inflammatory activities, contributing to its protective effects against a range of hepatotoxins, including heavy metals and alcohol [30]. Despite the promising therapeutic potential of these herbal compounds, their clinical use is fraught with limitations and challenges. One significant challenge is the variability in phytochemical content among different plant sources, which can lead to inconsistencies in efficacy and safety. Factors such as geographical location, cultivation methods, and harvesting times can significantly influence the concentration of active ingredients. This variability complicates the standardization of herbal products and can affect the reproducibility of clinical outcomes. Additionally, the potential side effects of herbal medications are often underreported, and some compounds may interact negatively with conventional pharmaceuticals. This raises concerns about the safety profiles of these natural products, especially in patients with pre-existing conditions or those taking multiple medications. The limitations of animal studies further complicate the situation, as findings from these models do not always accurately predict human outcomes. Factors such as differences in metabolism, absorption, and individual biological variability can result in discrepancies between animal and human responses to herbal treatments. In summary, while herbal antioxidants present a promising avenue for ameliorating xenobiotic-induced diseases, particularly drug-induced liver toxicity, their clinical assessment reveals significant limitations. The variability in phytochemical content, potential side effects, and the limitations of animal studies in predicting human outcomes all pose considerable challenges. A thorough understanding of these factors is crucial for the safe and effective incorporation of herbal medicines into clinical practice.

### Perspectives

There is a growing interest in developing plant-based therapies due to the natural antioxidant properties of plants. Plant extracts and bioactive compounds are increasingly utilized in biomedicine to mitigate the harmful effects of chemicals, specifically targeting oxidative stress as a crucial therapeutic pathway. We believe future research should prioritize establishing consistent concentrations of active compounds and determining safe dosages, as this will minimize variability in therapeutic effects and prevent potential toxicity. Thorough assessments of safety and toxicity are essential to identify adverse effects associated with long-term use or high doses of these supplements, given that excessive intake can pose risks. Additionally, investigating potential interactions between plant-derived supplements and prescription medications is crucial for ensuring safe use and maintaining treatment efficacy. Ongoing research should also aim to elucidate the mechanisms of action of plant-derived ingredients, effective dosages, and long-term outcomes to build a robust evidence base supporting their efficacy and safety. Considering the widespread exposure to various toxic substances in industrial and agricultural settings, we feel that exploring the combined effects of these chemical mixtures on human health is vital. Investigating plant-based interventions against individual toxicants alone may not capture the full scope of their impacts, underscoring the need to isolate and evaluate specific bioactive components for their effectiveness as pharmaceutical agents. In our view, addressing these research gaps can inspire new directions in the field, enhancing our understanding and utilization of plant-derived antioxidants to combat chemical toxicity effectively. This comprehensive approach will pave the way for the development of safe, effective health supplements that leverage the protective effects of nature.

## Figures and Tables

**Table 1:** Overview of natural products, their key active ingredients, and their protective effects.

Natural Product	Major Constituents	Effects	Refs.
Barley grass (*Hordeum leporinum*)	2-*O*-glycosylisovitexin (2-*O*-GIV; C_27_H_30_O_15_), flavone-C-glycosides, saponarin (C_27_H_30_O_15_), and lutonarin (C_27_H_30_O_16_)	Decreased lipid peroxidation and enhanced antioxidant capacity	[31,32]
Carrot (*Daucus Carota*)	Xanthophyll (C_40_H_56_O_2_), beta-carotene (C_40_H_56_), and other antioxidant carotenoids	Prevention of oxidative damage through strong antioxidant capacity	[31]
Citrus fruit	Flavonoids and vitamin C (C_6_H_8_O_6_)	Highly effective in protecting against oxidative damage	[33]
Eleuthero root (*Eleutherococcus senticosus*)	Four shogaols (bioactive compounds derived from ginger) with antioxidant properties	Provides strong protection against oxidative damage and lipid peroxidation	[34,35,36]
Ginger root (*Zingiber officinale*)	About 40 bioflavonoids, including proanthocyanidins and quercetin (C_15_H_10_O_7_)	Offers protection against oxidative damage and lipid peroxidation	[35,37,38]
Ginkgo leaf (*Ginkgo biloba*)	About 40 bioflavonoids, including proanthocyanidins and quercetin (C_15_H_10_O_7_)	Provides protection against oxidative damage while inhibiting inflammation and atherosclerosis	[33,35,39,40]
Grape (*Vitis* sp.) seed/skin	Antioxidant substances, over 90% proanthocyanidins	Efficient at preventing free radical formation and decreasing oxidative stress	[35]
Kudzu root (*Pueraria lobata*)	Isoflavone puerarin (C_21_H_20_O_9_, crude form more efficient)	Active against oxidative damage due to elevated antioxidant capacity	[41,42,43]
Milk thistle seed (*Silybum marianum*)	Silymarin (C_25_H_22_O_10,_ flavonoid complex: major constituent silybinin)	Provides protection against oxidative damage and lipid peroxidation; enhances antioxidant enzyme activity	[44,45,46,47]
Rosemary leaf (*Rosmarinus officinalis*)	Flavonoids such as cirsimarin (C_23_H_24_O_11_), diosmin (C_28_H_32_O_15_), hesperidin (C_28_H_34_O_15_), and homoplantaginin (C_22_H_22_O_11_)	Effectively protects against oxidative damage and also possesses analgesic properties	[31,48]
Schisandra fruit (*Schisandra chinensis*)	At least nine dibenzocyclooctene lignans	Provides protection against oxidative damage and helps preserve membrane integrity	[49]
Tomato (*Solanum lycopersicum*)	Lycopene (C_15_H_10_O_7_) and vitamin C (C_6_H_8_O_6_)	Offers strong protection against oxidative damage due to its high antioxidant content	[33]
Turmeric root (*Curcuma longa*)	Curcuminoids	Protects against oxidative damage and inflammation; inhibits various inflammatory molecules	[35]

**Table 2:** Overview of the interactions between some natural products and their effects on organ toxicity induced by environmental toxicants. The table lists the specific toxicant involved, the oxidative stress parameters observed, the resulting effects on the organism, and the natural products that can ameliorate these toxic effects.

Organ Toxicity	Environmental Toxicants	Oxidative Stress Parameters	Effects	Natural Products	Ref.
nephrotoxicity	Bromobenzene C_6_H_5_Br	increased LPO, reduced antioxidant enzyme activity	inflammation and irregularities in kidney function	withaferin A	[66]
reproductive and developmental toxicity	Lead Pb	redox imbalance	learning and memory impairments in young rats	allicin	[67]
reproductive and developmental toxicity	di-n-butyl phthalate C₁_6_H_22_O_4_	increased MDA, decreased SOD and GPx levels	multinucleated gonocytes and reduced serum testosterone levels	taxifolin	[68]
reproductive and developmental toxicity	ethanol C_2_H_5_OH	altered levels of reduced and oxidized glutathione	malformations and changes in embryonic behavior	24-epibrassinolide	[69]
nephrotoxicity	zinc oxide (ZnO) nanoparticles	reduced non-enzymatic antioxidant levels	reduced renal GSH, elevated serum inflammatory markers, and glucose levels	quercetin and arginine	[70]
nephrotoxicity	arsenic As	formation of 8-OxodG	raised methylation levels, urinary excretion	black raspberries	[71]
nephrotoxicity	arsenic As	reduced antioxidant enzyme activity, increased MDA	renal damage causing cellular apoptosis	thymoquinone	[72]
reproductive and developmental toxicity	arsenic As	regulation of Nfe2l2, StAR, Ppargc1a expressions	abnormalities in sperm and testis structure and function	ellagic and ferulic acid	[73]
reproductive and developmental toxicity	acrylamide C_3_H_5_NO	elevated ROS	mobility problems, egg development deficiencies	thymoquinone and curcumin	[74]
nephrotoxicity	rotenone C_23_H_22_O_6_	redox imbalance	renal dysfunction	catechin, quercetin, taxifolin	[75]
nephrotoxicity	cadmium Cd	decreased antioxidant enzyme activity, increased LPO	injury to proximal tubules, dysfunction of the glomerulus	thymoquinone	[76]
nephrotoxicity	cadmium Cd	decreased antioxidant enzyme activity, increased iNOS, MMP-9, pERK 1/2	alterations in tubular epithelium and glomerular structure	flavocoxid	[77]
reproductive and developmental toxicity	nicotine C_10_H_14_N_2_	reduced antioxidant enzyme levels	structural and functional impairment in the testis and brain	*Mentha spicata*	[78]

**Table 3:** Phytochemical composition of sweet potato (*Ipomoea batatas*) extracts, showing the total phenolic content (TPC), total flavonoid content (TFC), and individual flavonoid contents (rutin and myricetin), as well as caffeic acid levels in both ethyl acetate and methanol extracts. Values are expressed in micrograms per milligram of dry extract, indicating the bioactive potential of the extracts.

Phytochemical Content	Content (Dry Extract)	±
Total Phenolic (TPC)	304.32 μg gallic acid equivalents/mg	7.20
Total Flavonoid (TFC)	214.77 μg quercetin equivalents/mg	4.09
Rutin	7.3 μg/mg	0.12
Myricetin	2.7 μg/mg	0.14
Caffeic Acid (ethyl acetate extract)	4.05 μg/mg	0.22
Caffeic Acid (methanol extract)	1.92 μg/mg	0.17

**Table 4:** Examples of natural products mentioned in this section that were tested for their protective effects in various experimental models, detailing the experimental setup, observed results, and underlying mechanisms of action.

Natural Product	Experimental Model	Experimental Details and Results	Protective Effects and Mechanisms	Ref.
*Boerhavia diffusa*	*Drosophila larvae*	*B. diffusa* aqueous extract (BDAE) was tested at four concentrations: 25, 50, 100, and 200 mg/mL. Results: BDAE mitigated toluene-induced developmental and reproductive toxicity, significantly improved reproductive and developmental parameters, and increased antioxidant enzyme levels.	Effectiveness: Reduced developmental and reproductive toxicity induced by toluene. Mechanism: Antioxidant activity, elevated antioxidant enzyme levels (catalase, glutathione-S-transferase, superoxide dismutase).	[81]
Isoliquiritigenin (ISL)	Zebrafish embryos	ISL was used at 4 μM with exposure to BDE47 (1 and 10 μM) for up to 120 h post-fertilization. Results: ISL mitigated BDE47-induced developmental abnormalities, excessive ROS accumulation, and altered apoptosis-related gene expressions.	Effectiveness: Ameliorated developmental toxicity caused by BDE47. Mechanism: Enhanced antioxidant enzyme activities (SOD and CAT), modulated apoptosis-related gene expressions (p53, Bcl-2, caspase 3, caspase 9).	[82]
*Calliandra portoricensis* (CP)	Rats	CP was evaluated for its effects against ovarian and uterine toxicity induced by benzo[a]pyrene (BaP) and N-methyl nitrosourea (NMU). Results: CP reduced toxic responses from NMU and BaP, alleviating oxidative damage and inflammation.	Effectiveness: Mitigated ovarian and uterine toxicity from carcinogens. Mechanism: Antioxidant and anti-inflammatory properties.	[85]
*Ipomoea batatas*	Male Sprague Dawley rats	*Ipomoea batatas* extracts were tested for protective effects against BPA-induced gonadotoxicity at 300 mg/kg. Results: Enhanced sexual behavior, improved semen quality, increased levels of testosterone, FSH, LH, and estradiol, and restored antioxidant enzyme levels.	Effectiveness: Improved reproductive health and mitigated BPA-induced toxicity. Mechanism: Antioxidant activity, restoration of hormonal balance, improved semen quality.	[85]
Beetroot (*Beta vulgaris* L.)	Mice	Beetroot was evaluated for its protective effects against chlorpyrifos (CPF) nephrotoxicity. Results: Restored antioxidant levels and reduced oxidative stress markers.	Effectiveness: Mitigated CPF-induced nephrotoxicity. Mechanism: Enhanced antioxidant levels, reduced oxidative stress. Beetroot extracts enhance nuclear factor erythroid 2-related factor 2 (Nrf2), which is essential for promoting the expression of defensive enzymes.	[85]
*Opuntia ficus indica*	Mice	Opuntia cladodes extract was tested for its effects on liver damage caused by chlorpyrifos. Results: Improved liver function, reduced oxidative stress, and normalized biochemical parameters.	Effectiveness: Protected against liver damage from CPF. Mechanism: Restored antioxidant levels, reduced oxidative stress and liver damage.	[85]

## Data Availability

No data available.

## Abbreviations

8-OxodG	8-hydroxy-2′-deoxyguanosine
ALT	Alanine transaminase
ALP	Alkaline phosphatase
AST	Aspartate aminotransferase
ATPase	Adenosine triphosphatase
BDE47	2,2′,4,4′-Tetrabromodiphenyl ether
BPA	Bisphenol A
BUN	Blood urea nitrogen
CAT	Catalase
COX	Cyclooxygenase
CP	*Calliandra portoricensis*
CPF	Chlorpyrifos
CYP	Cytochrome P450
DMF	Dimethylformamide
EC10	Effective concentration 10%
EC50	Effective concentration 50%
EC90	Effective concentration 90%
GPx	Glutathione peroxidase
GSH	Glutathione
GST	Glutathione S-transferase
HPTLC	High-performance thin layer chromatography
Hsp	Heat shock protein
ISL	Isoliquiritigenin
LC15	Lethal concentration 15%
LC50	Lethal concentration 50%
LC85	Lethal concentration 85%
LOX	Lipoxygenase
LPO	Lipid Peroxidation
MDA	Malondialdehyde
MMP	Mitochondrial membrane potential
mROS	Mitochondrial reactive oxygen species
mRNA	Messenger RNA
Nfe2l2	Nuclear factor erythroid 2-related factor 2
NO	Nitric oxide
NOX	NADPH oxidase
Nrf2	Nuclear factor erythroid 2-related factor 2
PDGF	Platelet-derived growth factor
PI3K	Phosphoinositide-3-kinase
POD	Peroxidase
RNS	Reactive nitrogen species
ROS	Reactive oxygen species
SOD	Superoxide dismutase
StAR	Steroidogenic acute regulatory protein
TAC	Total antioxidant capacity
TFAM	Mitochondrial transcription factor A
TTH	Total thiol content
TSH	Thyroid-stimulating hormone
XO	Xanthine oxidase
